# A Novel Nomogram Based on Machine Learning-Pathomics Signature and Neutrophil to Lymphocyte Ratio for Survival Prediction of Bladder Cancer Patients

**DOI:** 10.3389/fonc.2021.703033

**Published:** 2021-06-17

**Authors:** Siteng Chen, Liren Jiang, Encheng Zhang, Shanshan Hu, Tao Wang, Feng Gao, Ning Zhang, Xiang Wang, Junhua Zheng

**Affiliations:** ^1^ Department of Urology, Shanghai General Hospital, Shanghai Jiao Tong University School of Medicine, Shanghai, China; ^2^ Department of Pathology, Shanghai General Hospital, Shanghai Jiao Tong University School of Medicine, Shanghai, China; ^3^ Department of Pharmacy, Zhujiang Hospital, Southern Medical University, Guangzhou, China; ^4^ Department of Clinical Pharmacy, Shanghai General Hospital, Shanghai Jiao Tong University School of Medicine, Shanghai, China; ^5^ Department of Urology, Ruijin Hospital, Shanghai Jiao Tong University School of Medicine, Shanghai, China

**Keywords:** bladder cancer, pathomics, machine learning, neutrophil to lymphocyte ratio, prognosis

## Abstract

Traditional histopathology performed by pathologists through naked eyes is insufficient for accurate survival prediction of bladder cancer (BCa). In addition, how neutrophil to lymphocyte ratio (NLR) could be used for prognosis prediction of BCa patients has not been fully understood. In this study, we collected 508 whole slide images (WSIs) of hematoxylin–eosin strained BCa slices and NLR value from the Shanghai General Hospital and The Cancer Genome Atlas (TCGA), which were further processed for nuclear segmentation. Cross-verified prediction models for predicting clinical prognosis were constructed based on machine learning methods. Six WSIs features were selected for the construction of pathomics-based prognosis model, which could automatically distinguish BCa patients with worse survival outcomes, with hazard ratio value of 2.19 in TCGA cohort (95% confidence interval: 1.63–2.94, p <0.0001) and 3.20 in General cohort (95% confidence interval: 1.75–5.87, p = 0.0014). Patients in TCGA cohort with high NLR exhibited significantly worse clinical survival outcome when compared with patients with low NLR (HR = 2.06, 95% CI: 1.29–3.27, p <0.0001). External validation in General cohort also revealed significantly poor prognosis in BCa patients with high NLR (HR = 3.69, 95% CI: 1.83–7.44 p <0.0001). Univariate and multivariate cox regression analysis proved that both the MLPS and the NLR could act as independent prognostic factor for overall survival of BCa patients. Finally, a novel nomogram based on MLPS and NLR was constructed to improve their clinical practicability, which had excellent agreement with actual observation in 1-, 3- and 5-year overall survival prediction. Decision curve analyses both in the TCGA cohort and General cohort revealed that the novel nomogram acted better than both the tumor grade system in prognosis prediction. Our novel nomogram based on MLPS and NLR could act as an excellent survival predictor and provide a scalable and cost-effective method for clinicians to facilitate individualized therapy. Nevertheless, prospective studies are still needed for further verifications.

## Background

Bladder cancer (BCa) is one of the most common malignant tumors worldwide. It is estimated that there will be 83,730 new cases of BCa and 17,200 BCa-related deaths in the United States in 2021 (1). Although more than 75% of BCa patients are without muscle invasion, up to 10 to 15% of them could still progress to muscle-invasive disease after initial surgical treatment (2, 3), which results in poor clinical outcomes. In addition, about 10–15% of initially diagnosed BCa patients have metastatic lesion, suffered from a survival possibility less than 5% in five-year (4). Therefore, it is of great urgence to find out useful predictors for predicting the clinical outcomes of BCa patients.

As an emerging high-throughput process of medical images, pathomics combines artificial intelligence and digitalized pathology, which displays its blueprint in future pathology diagnosis (5, 6). The digitization in whole slide image (WSI) shows the advantage in artificial intelligence based pathological diagnosis as they provide non-manually handled specimen images (7). In addition, the neutrophil-to-lymphocyte ratio (NLR) has also been reported to be a valid biomarker for prognosis of multiple malignancies (8–10), including urothelial carcinoma (11). A systemic inflammatory marker score was also proved to be an effective predictor for tumor recurrence and progression of BCa without muscular invasion (12). However, how pathomics and NLR could be expediently used for prognosis prediction of BCa patients in clinical practice has not been fully understood.

In this study, we firstly carried out machine learning methods based on WSI to investigate the prognostic value of pathomics signature and NLR in BCa patients. Subsequently, we constructed and verified a novel nomogram based on pathomics signature and NLR to explore convenient and effective ways for prognosis prediction of BCa patients in clinical practice.

## Methods

### Patient Cohorts and Data Resource

Our patient cohorts come from two independent data source—Shanghai General Hospital and The Cancer Genome Atlas (TCGA,https://portal.gdc.cancer.gov). We recruited 102 BCa patients, who received operative treatment from January 2009 to December 2016 in the Shanghai General Hospital (General cohort). All the included patients shall meet the following inclusion criteria: (i) underwent radical or partial cystectomy, without preoperative treatment and positive residual tumor margin; (ii) diagnosed as a single type of primary malignant bladder tumor with pathological evidence; (iii) with complete clinicopathologic data and clinical follow-up information; (iv) with access to total neutrophil count and total lymphocyte count of peripheral blood before surgery; and (v) with access to corresponding hematoxylin–eosin (H&E) staining tumor slides.

Another 406 BCa patients meeting the first two criteria mentioned above and with open-access histopathology images from TCGA were also enrolled in this study. Detailed clinical information and RNA sequencing data was also acquired from TCGA database. RNA sequencing data was normalized using the RSEM method (13). Genes with transcriptomic value less than 70% of the total samples were eliminated for further analysis. Clinical characteristics of BCa patients recruited in this study were shown in **Table 1**.

**Table 1:** Basic clinical characteristics of patients in the TCGA cohort and General cohort.

	TCGA Cohort (406)	General Cohort (102)
**Gender**		
Male	299 (73.6%)	88 (86.3%)
Female	107 (26.4%)	14 (13.7%)
**Age(years)**		
≥65	255 (62.8%)	62 (60.8%)
<65	151 (37.2%)	40 (39.2%)
**Race**		
White	323 (79.5%)	0
Asian	43 (10.6%)	102 (100%)
Black	23 (5.7%)	0
Unknown	17 (4.2%)	0
**Grade**		
High	383 (94.3%)	94 (92.2%)
Low	20 (4.9%)	8 (7.8%)
Unknown	3 (0.8%)	0
**Stage**		
I	2 (0.5%)	0
II	129 (31.8%)	57 (55.9%)
III	140 (34.5%)	23 (22.5%)
IV	133 (32.7%)	22 (21.6%)
Unknown	2 (0.5%)	0
**Survival status**		
Alive	227 (55.9%)	61 (58.7%)
Dead	179 (44.1%)	43 (41.3%)

TCGA, The Cancer Genome Atlas.

### Whole Slide Image Process and Analysis Pipeline

Raw H&E profiles of WSI without color deconvolution or any watershed processing were segmented into tiles. We eliminated tiles with non-cell objects or excess whitespace. The eligible tiles were further scanned and detected *via* QuPath digital pathology software (14) to construct modules for nuclear segmentation. Nuclear segmentation was carried out for recognized objects through Watershed cell detection based on segmentation parameters (15). A serious of tiles from the same H&E image were further reconstituted for representation of the original WSI. The detected image factors were shown in **Table S1**.

**Figure 1:**
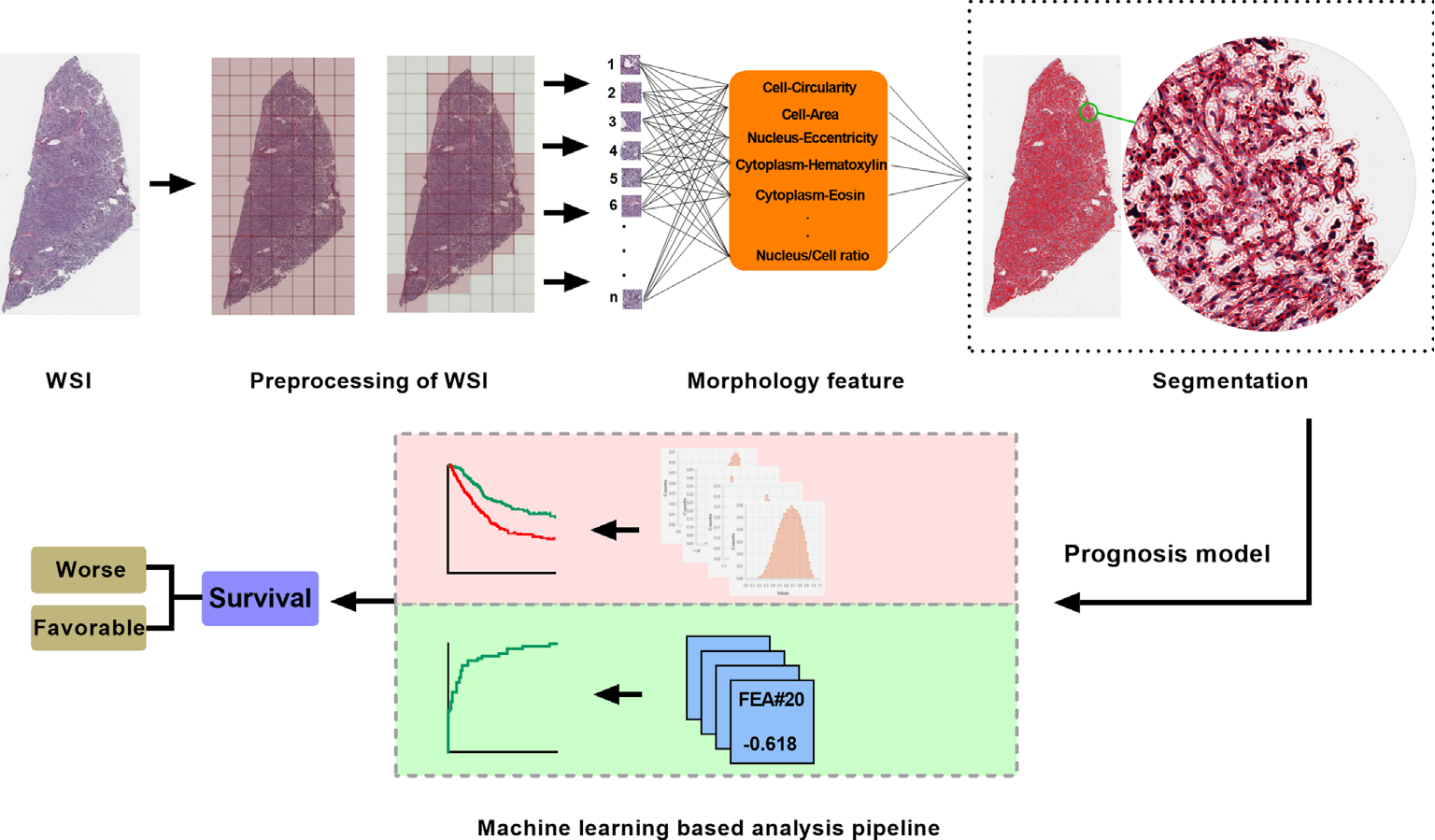
The workflow of histopathology image processing and machine learning analysis in this study. WSI, whole slide image.

We built an analysis pipeline based on machine learning algorithm to intelligently analyze the detected H&E image features for different clinical applications. Least absolute shrinkage and selection operator *via glmnet* package (16) were used to identify optimal digital pathological features and calculate coefficients of each features in pathomics-based models. The workflow of histopathology image processing and analysis pipeline was shown in **Figure 1**.

### Neutrophil to Lymphocyte Ratio

NLR was defined as the total neutrophil count divided by the total lymphocyte count (9). For the patients from the Shanghai General Hospital, the total neutrophil count and the total lymphocyte count of peripheral blood were tested before surgery. For the patients in the TCGA cohort, the total neutrophil count and the total lymphocyte count were estimated from transcriptomic data by using CIBERSORT based on the abundances of 22 types of immune cell (17).

### Statistical Analysis

In this study, Statistical Package for Social Sciences 24.0 software (SPSS Inc., Chicago, IL, USA) and R 3.6.2 were used to conduct data analyses. Kaplan–Meier (KM) curve analysis with hazard ratio (HR) and 95% confidence interval (CI) were carried out to identify different survival outcomes. The prognostic nomogram was established based on MLPS and NLR *via* the *rms* and *nomogramEx* packages in R, which was evaluated *via* Calibration and decision curve. The cut-off value of each prognostic biomarker in different patient cohort was set as the optional value defined through *survminer* packages in R.

## Results

### Developed and Verified the Machine Learning-Based Pathomics Signature for BCa

**Figure 2:**
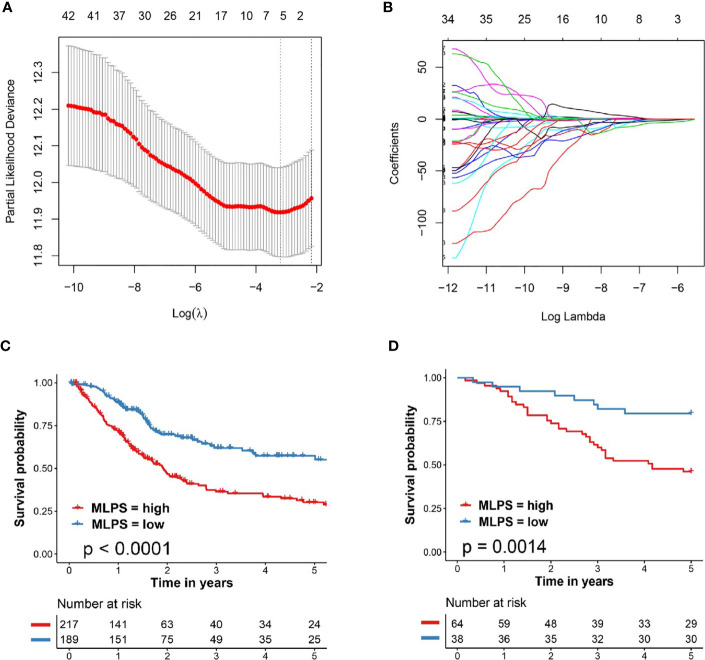
Developed and verified the pathomics-based prognosis model for BCa. **(A, B)** The tenfold cross-validated error and the profile of coefficients in the model at varying levels of penalization plotted against the log (lambda) sequence for least absolute shrinkage and selection operator analysis. **(C)** Kaplan–Meier survival analysis of overall survival predicted by pathomics-based prognosis model for BCa patients in the TCGA cohort. **(D)** Kaplan–Meier survival analysis of overall survival predicted by pathomics-based prognosis model for BCa patients in the validation cohort (General cohort). BCa, bladder cancer; TCGA, The Cancer Genome Atlas; MLPS, machine learning-based pathomics signature.

As shown in **Figure 2A**, the left vertical line which equaled to the minimum tenfold cross-validated error arrived at 6, indicating that six image factors were screened out to be the most prognostic factors for patients with BCa. The selected image factors included Nucleus/Cell area ratio, Nucleus circularity, Cell hematoxylin OD std dev, Cell area, Cell mincaliper, Cell eosin OD min.

The regression coefficients (β) of each selected image factors were also extracted from the LASSO analysis in **Figure 2B** (β_Nucleus/Cell area ratio_ = −3.831038781, β_Nucleus circularity_ = −2.515644503, β_Cell hematoxylin OD std dev_ = −0.915105249, β_Cell area_ = 0.003702581, β_Cell mincaliper_ = 0.012840928, β_Cell eosin OD min_ = 0.958474254). The machine learning-based pathomics signature (MLPS) was then established as follows: MLPS = Nucleus/Cell area ratio ∗ (−3.831038781) + Nucleus circularity ∗ (−2.515644503) + Cell hematoxylin OD std dev ∗ (−0.915105249) + Cell area ∗ (0.003702581) + Cell mincaliper ∗ (0.012840928) + Cell eosin OD min ∗ (0.958474254).

We further evaluated the performance of our pathomics-based prognosis model in BCa patients through KM curve survival analysis. As shown in **Figure 2C**, patients in the TCGA cohort with high MLPS exhibited significantly worse clinical survival outcome when compared with patients with low MLPS (HR = 2.19, 95% CI: 1.63–2.94, p <0.0001). External validation in BCa patients from Shanghai General Hospital (General cohort) also demonstrated notably poor prognosis in BCa patients with high MLPS (HR = 3.20, 95% CI: 1.75–5.87, p = 0.0014, **Figure 2D**), indicating the forceful performance of the pathomics-based prognosis model for BCa patients.

### Important Role of NLR in Clinical Prognosis of Patients With BCa

**Figure 3:**
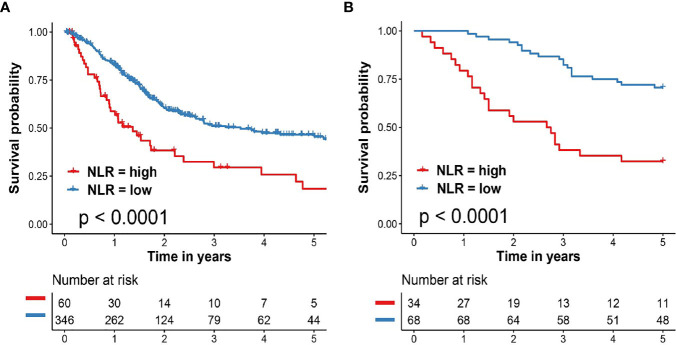
Important role of NLR in clinical prognosis of patients with BCa. **(A)** Kaplan–Meier survival analysis of overall survival predicted by NLR for BCa patients in the TCGA cohort. **(B)** Kaplan–Meier survival analysis of overall survival predicted by NLR for BCa patients in the validation cohort (General cohort). BCa, bladder cancer; TCGA, The Cancer Genome Atlas; NLR, neutrophil to lymphocyte ratio.

We next to carry out KM curve survival analysis to identify the important role of NLR in clinical prognosis of BCa patients. As shown in **Figure 3A**, patients in the TCGA cohort with high NLR exhibited significantly worse clinical survival outcome when compared with patients with low NLR (HR = 2.06, 95% CI: 1.29–3.27, p <0.0001). External validation in General cohort also revealed significantly poor prognosis in BCa patients with high NLR (HR = 3.69, 95% CI: 1.83–7.44 p <0.0001, **Figure 3B**).

To further evaluate the important roles of MLPS and NLR for BCa patients, we performed univariate and multivariate Cox regression analysis in two different patient cohorts. We find out that both the MLPS and the NLR could act as independent prognostic factor for overall survival of patients with BCa (**Table 2**).

**Table 2:** Univariate and multivariate cox regression analysis of prognostic markers two independent patient cohorts.

Cohort		Univariate			Multivariate		
		HR	95% CI	p	HR	95% CI	p
**TCGA**	**Sex**						
	male *vs* female	0.87	0.63–1.20	0.379	**/**	**/**	**/**
	**Age**						
	≥65 *vs* <65	1.98	1.41–2.78	<0.0001	1.77	1.26–2.50	0.001
	**Grade**						
	high *vs* low	2.95	0.73–11.96	0.128	**/**	**/**	**/**
	**Stage**						
	III/IV *vs* I/II	2.16	1.50–3.12	<0.0001	1.86	1.28–2.69	0.001
	**MLPS**						
	high *vs* low	2.20	1.61–3.01	<0.0001	1.93	1.39–2.66	<0.0001
	**NLR**						
	high *vs* low	2.06	1.44–2.96	<0.0001	1.55	1.06–2.25	0.023
**General**	**Sex**						
	male *vs* female	0.88	0.39–1.98	0.755	**/**	**/**	**/**
	**Age**						
	≥65 *vs* <65	1.62	0.84–3.10	0.149	**/**	**/**	**/**
	**Grade**						
	high *vs* low	23.72	0.30–>100	0.155	**/**	**/**	**/**
	**Stage**						
	III/IV *vs* I/II	3.04	1.63–5.65	<0.0001	2.63	1.39–4.97	0.003
	**MLPS**						
	high *vs* low	3.22	1.49–6.95	0.003	2.78	1.27–6.09	0.01
	**NLR**						
	high *vs* low	3.75	2.05–6.86	<0.0001	2.71	1.45–5.06	0.002

TCGA, The Cancer Genome Atlas; HR, hazard ratio; CI, confidence interval.

### Construction and Evaluation of a Novel Nomogram Based on MLPS and NLR

**Figure 4:**
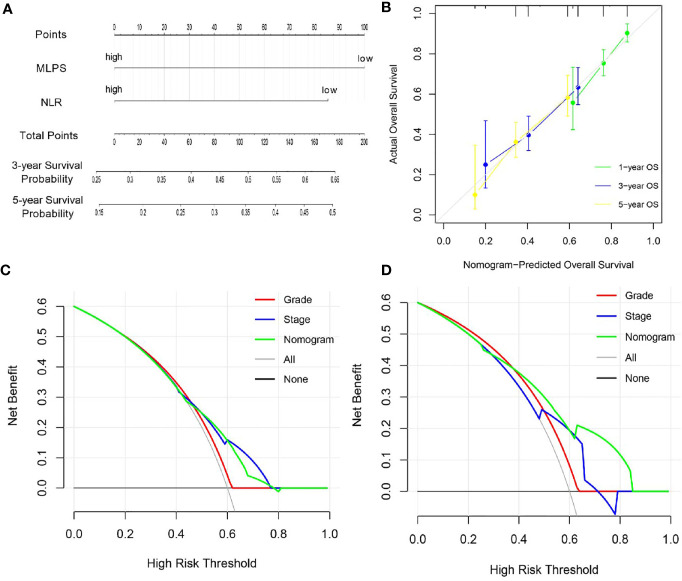
Construction and evolution of a prognostic nomogram based on MLPS and NLR. **(A)** Nomogram based on MLPS and NLR for BCa patients in the TCGA cohort. **(B)** Calibrate plot evaluating the nomogram-predicted probabilities of 1-, 3- and 5-years survival with the actual overall survival. **(C, D)** Decision curve analyses comparing overall survival benefits among the nomogram, tumor grade and stage in the TCGA cohort and the General cohort, respectively. MLPS, machine learning-based pathomics signature; NLR, neutrophil to lymphocyte ratio; BCa, bladder cancer; TCGA, The Cancer Genome Atlas.

Since MLPS and NLR had been proved to be independent prognostic factors for BCa patients based on the univariate and multivariate Cox regression algorithm, we further tried to construct a novel nomogram based on MLPS and NLR to improve their clinical practicability (**Figure 4A**). The calibration plots revealed that 1-, 3- and 5-year OS probability predicted by the integrated nomogram model had excellent agreement with actual observation (**Figure 4B**), indicating good ability to accurately predict OS status for BCa. Further decision curve analysis in the TCGA cohort revealed that when the threshold probability was larger than 0.42, using the novel nomogram for OS prediction added more benefit than tumor grade system (**Figure 4C**). Furthermore, verification of decision curve analysis in General cohort indicated that the novel nomogram acted better than both the tumor grade and stage system in prognosis prediction (**Figure 4D**), indicating that the nomogram was clinically useful.

## Discussion

Artificial intelligence revolutionizes the traditional healthcare system in various areas including radiology and pathology (5, 18). Pathomics and radiomics, the applications of artificial intelligence, belong to high-throughput omics and show the eligibility in malignancy diagnosis and prediction (6, 19). In addition, cooperated with machine learning methods, pathomics has exhibited its eligibility in pathological diagnosis, including lung cancers, breast cancers, neuron cancers and skin cancers, with very high accuracy (20–23).

In this study, we firstly established and verified a pathomics-based prognosis model from WSI for predicting the survival status of BCa patients. Our prognosis model showed remarkable performance in distinguishing BCa patients with high survival risk in both two independent cohorts, indicating its potential in predicting the prognosis of BCa patients. Malignancies with polytypic nuclei, high nucleoplasm ratio and hyperchromatic nuclei usually stand for higher grade of pathological classification and worse prognosis (24). Hence, in this study, the unrevealed implications for BCa prognosis prediction might be based on classic pathological theories.

Through the currently outbreaking research on tumor immune system and tumor microenvironment, we start to realize that tumor immune response may be adjusted by tumor progression and afterwards affect tumor growth (25, 26). In addition, focal tumor immune responses show possible association with the systemic immune responses in cancer patients (25, 27). Studies show that changes in systemic inflammation environment, such as NLR, can be a useful biomarker for predicting the survival of cancer patients (25, 28, 29).

As reported previously, neutrophil can promote tumor progression through changing the tumor environment (28, 30). Whereas lymphocytes, especially CD8 positive T cells, are the main forces to suppressing and removing tumor cells (28, 31). The important roles of inflammatory markers in urothelial carcinoma have also been gradually recognized. The combination of preoperative NLR, C-reactive protein, and plasma fibrinogen could act as an effective predictor for prognosis of patient with upper tract urothelial carcinoma (32). In addition, pretreatment NLR was proved to be associated with advanced tumor stage and increased cancer-specific mortality in BCa patients receiving radical cystectomy (33).

Here, we constructed and evaluated a novel nomogram based on MLPS and NLR for BCa patients. The MLPS based on WSIs contains various important pathologic features, including nucleus/cell area ratio, nucleus circularity, and cell area. The NLR value can reflect the systemic immune response background, which is detected from peripheral blood. The combination analysis of MLPS and NLR can improve the unilateral prognostic analysis and hence increase the prognostic accuracy. Intriguingly, considering the NLR can predict traditional chemotherapy outcomes in BCa patients (34), the integrated nomogram might show the potential for further drug resistance prediction.

Limitations could also be found in this study. Firstly, only 43 pathological signatures were detected from the segmented tile of each WSI, which reflects the need of more robust segmentation methods. Secondly, our study is retrospective and may be subject to inherent biases, although we have verified our major results in two independent patient cohorts. The machine learning-based models still need further verifications from prospective studies.

## Conclusion

In conclusion, we identified the important roles of MLPS and NLR in the prognosis prediction of patients with BCa. The novel prognostic nomogram based on MLPS and NLR was further constructed and evaluated to act as an excellent survival predictor and provide a scalable and cost-effective method for clinicians to facilitate individualized therapy. Nevertheless, prospective studies are still needed for further verifications.

## Data Availability Statement

The raw data supporting the conclusions of this article will be available from the authors upon reasonable request.

## Ethics Statement

The studies involving human participants were reviewed and approved by the Research Ethics Committee of Shanghai General Hospital. The patients/participants provided their written informed consent to participate in this study.

## Author Contributions

JZ, XW and NZ designed the study. SC, TW, FG and EZ acquired the data. SC, LJ and SH analyzed the data. SC and NZ wrote the report, which was edited by all authors. JZ and XW supervised the project. All authors contributed to the article and approved the submitted version.

## Funding

This work was supported by the National Natural Science Foundation of China (81972393 and 82002665).

## Conflict of Interest

The authors declare that the research was conducted in the absence of any commercial or financial relationships that could be construed as a potential conflict of interest.

## References

[1] Siegel RL Miller KD Fuchs HE Jemal A. Cancer Statistics, 2021. CA Cancer J Clin (2021) 71:7–33. 10.3322/caac.21654 33433946

[2] Abufaraj M Dalbagni G Daneshmand S Horenblas S Kamat AM Kanzaki R. The Role of Surgery in Metastatic Bladder Cancer: A Systematic Review. Eur Urol (2018) 73:543–57. 10.1016/j.eururo.2017.09.030 PMC8177016 29122377

[3] Millán-Rodríguez F Chéchile-Toniolo G Salvador-Bayarri J Palou J Vicente-Rodríguez J. Multivariate Analysis of the Prognostic Factors of Primary Superficial Bladder Cancer. J Urol (2000) 163:73–8. 10.1016/s0022-5347(05)67975-x 10604317

[4] Stein JP Lieskovsky G Cote R Groshen S Feng AC Boyd S. Radical Cystectomy in the Treatment of Invasive Bladder Cancer: Long-Term Results in 1,054 Patients. J Clin Oncol (2001) 19:666–75. 10.1200/jco.2001.19.3.666 11157016

[5] Schuettfort VM Pradere B Rink M Comperat E Shariat SF. Pathomics in Urology. Curr Opin Urol (2020) 30:823–31. 10.1097/MOU.0000000000000813 32881725

[6] Cao R Yang F Ma SC Liu L Zhao Y Li Y. Development and Interpretation of a Pathomics-Based Model for the Prediction of Microsatellite Instability in Colorectal Cancer. Theranostics (2020) 10:11080–91. 10.7150/thno.49864 PMC7532670 33042271

[7] Yu H Gao F Jiang L. S. Ma. Development of a Whole Slide Imaging System on Smartphones and Evaluation With Frozen Section Samples. JMIR Mhealth Uhealth (2017) 5:e132. 10.2196/mhealth.8242 28916508 PMC5622289

[8] Akinci Ozyurek B Sahin Ozdemirel T Buyukyaylaci Ozden S Erdogan Y Kaplan B. T. Kaplan. Prognostic Value of the Neutrophil to Lymphocyte Ratio (NLR) in Lung Cancer Cases. Asian Pac J Cancer Prev (2017) 18:1417–21. 10.22034/apjcp.2017.18.5.1417 PMC5555556 28612596

[9] Shirasawa M Yoshida T Horinouchi H Kitano S Arakawa S Matsumoto Y. Prognostic Impact of Peripheral Blood Neutrophil to Lymphocyte Ratio in Advanced-Stage Pulmonary Large Cell Neuroendocrine Carcinoma and its Association With the Immune-Related Tumour Microenvironment. Br J Cancer (2021) 124:925–32. 10.1038/s41416-020-01188-7 PMC7921668 33250511

[10] Szkandera J Gerger A Liegl-Atzwanger B Absenger G Stotz M Friesenbichler J. The Lymphocyte/Monocyte Ratio Predicts Poor Clinical Outcome and Improves the Predictive Accuracy in Patients With Soft Tissue Sarcomas. Int J Cancer (2014) 135:362–70. 10.1002/ijc.28677 24347236

[11] Lucca I Jichlinski P Shariat SF Rouprêt M Rieken M Kluth LA. The Neutrophil-to-lymphocyte Ratio as a Prognostic Factor for Patients With Urothelial Carcinoma of the Bladder Following Radical Cystectomy: Validation and Meta-Analysis. Eur Urol Focus (2016) 2:79–85. 10.1016/j.euf.2015.03.001 28723455

[12] Cantiello F Russo GI Vartolomei MD Farhan ARA Terracciano D Musi G. Systemic Inflammatory Markers and Oncologic Outcomes in Patients With High-Risk Non-Muscle-Invasive Urothelial Bladder Cancer. Eur Urol Oncol (2018) 1:403–10. 10.1016/j.euo.2018.06.006 31158079

[13] Li B Dewey CN. RSEM: Accurate Transcript Quantification From RNA-Seq Data With or Without a Reference Genome. BMC Bioinf (2011) 12:323. 10.1186/1471-2105-12-323 PMC3163565 21816040

[14] Bankhead P Loughrey MB Fernández JA Dombrowski Y McArt DG Dunne PD. Qupath: Open Source Software for Digital Pathology Image Analysis. Sci Rep (2017) 7:16878. 10.1038/s41598-017-17204-5 29203879 PMC5715110

[15] Kulkarni PM Robinson EJ Sarin Pradhan J Gartrell-Corrado RD Rohr BR Trager MH. Deep Learning Based on Standard H&E Images of Primary Melanoma Tumors Identifies Patients at Risk for Visceral Recurrence and Death. Clin Cancer Res (2020) 26:1126–34. 10.1158/1078-0432.Ccr-19-1495 PMC8142811 31636101

[16] Friedman J Hastie T Tibshirani R. Regularization Paths for Generalized Linear Models Via Coordinate Descent. J Stat Softw (2010) 33:1–22. 20808728 PMC2929880

[17] Newman AM Steen CB Liu CL Gentles AJ Chaudhuri AA Scherer F. Determining Cell Type Abundance and Expression From Bulk Tissues With Digital Cytometry. Nat Biotechnol (2019) 37:773–82. 10.1038/s41587-019-0114-2 PMC6610714 31061481

[18] Jiang Y Jin C Yu H Wu J Chen C Yuan Q. Development and Validation of a Deep Learning CT Signature to Predict Survival and Chemotherapy Benefit in Gastric Cancer: A Multicenter, Retrospective Study. Ann Surg (2020). 10.1097/sla.0000000000003778 31913871

[19] Chen ST Zhang N Jiang LR Gao F Shao JL Wang T. Clinical Use of a Machine Learning Histopathological Image Signature in Diagnosis and Survival Prediction of Clear Cell Renal Cell Carcinoma. Int J Cancer (2021) 148:780–90. 10.1002/ijc.33288 32895914

[20] Yu KH Zhang C Berry GJ Altman RB Re C Rubin DL. Predicting non-Small Cell Lung Cancer Prognosis by Fully Automated Microscopic Pathology Image Features. Nat Commun (2016) 7:12474. 10.1038/ncomms12474 27527408 PMC4990706

[21] Ehteshami Bejnordi B Veta M Johannes van Diest P van Ginneken B Karssemeijer N Litjens G. Diagnostic Assessment of Deep Learning Algorithms for Detection of Lymph Node Metastases in Women With Breast Cancer. JAMA (2017) 318:2199–210. 10.1001/jama.2017.14585 PMC5820737 29234806

[22] Kickingereder P Isensee F Tursunova I Petersen J Neuberger U Bonekamp D. Automated Quantitative Tumour Response Assessment of MRI in Neuro-Oncology with Artificial Neural Networks: A Multicentre, Retrospective Study. Lancet Oncol (2019) 20:728–40. 10.1016/s1470-2045(19)30098-1 30952559

[23] Esteva A Kuprel B Novoa RA Ko J Swetter SM Blau HM. Dermatologist-Level Classification of Skin Cancer With Deep Neural Networks. Nature (2017) 542:115–8. 10.1038/nature21056 PMC8382232 28117445

[24] Humphrey PA Moch H Cubilla AL Ulbright TM Reuter VE. The 2016 WHO Classification of Tumours of the Urinary System and Male Genital Organs-Part B: Prostate and Bladder Tumours. Eur Urol (2016) 70:106–19. 10.1016/j.eururo.2016.02.028 26996659

[25] Templeton AJ McNamara MG Seruga B Vera-Badillo FE Aneja P Ocana A. Prognostic Role of Neutrophil-to-Lymphocyte Ratio in Solid Tumors: A Systematic Review and Meta-Analysis. J Natl Cancer Inst (2014) 106:dju124. 10.1093/jnci/dju124 24875653

[26] Grivennikov SI Greten FR Karin M. Immunity, Inflammation, and Cancer. Cell (2010) 140:883–99. 10.1016/j.cell.2010.01.025 PMC2866629 20303878

[27] Aggarwal BB Vijayalekshmi RV Sung B. Targeting Inflammatory Pathways for Prevention and Therapy of Cancer: Short-Term Friend, Long-Term Foe. Clin Cancer Res (2009) 15:425–30. 10.1158/1078-0432.CCR-08-0149 19147746

[28] Kang J Chang Y Ahn J Oh S Koo DH Lee YG. Neutrophil-to-Lymphocyte Ratio and Risk of Lung Cancer Mortality in a Low-Risk Population: A Cohort Study. Int J Cancer (2019) 145:3267–75. 10.1002/ijc.32640 31454064

[29] Kawahara T Kato M Tabata K Kojima I Yamada H Kamihira O. A High Neutrophil-to-Lymphocyte Ratio Is a Poor Prognostic Factor for Castration-Resistant Prostate Cancer Patients Who Undergo Abiraterone Acetate or Enzalutamide Treatment. BMC Cancer (2020) 20:919. 10.1186/s12885-020-07410-2 32977754 PMC7519532

[30] Schaider H Oka M Bogenrieder T Nesbit M Satyamoorthy K Berking C. Differential Response of Primary and Metastatic Melanomas to Neutrophils Attracted by IL-8. Int J Cancer (2003) 103:335–43. 10.1002/ijc.10775 12471616

[31] Taniuchi I. Cd4 Helper and CD8 Cytotoxic T Cell Differentiation. Annu Rev Immunol (2018) 36:579–601. 10.1146/annurev-immunol-042617-053411 29677476

[32] Tanaka N Kikuchi E Kanao K Matsumoto K Shirotake S Miyazaki Y. Impact of Combined Use of Blood-Based Inflammatory Markers on Patients With Upper Tract Urothelial Carcinoma Following Radical Nephroureterectomy: Proposal of a Cumulative Marker Score as a Novel Predictive Tool for Prognosis. Eur Urol Focus (2015) 1:54–63. 10.1016/j.euf.2015.02.001 28723357

[33] Viers BR Boorjian SA Frank I Tarrell RF Thapa P Karnes RJ. Pretreatment Neutrophil-to-Lymphocyte Ratio Is Associated With Advanced Pathologic Tumor Stage and Increased Cancer-Specific Mortality Among Patients With Urothelial Carcinoma of the Bladder Undergoing Radical Cystectomy. Eur Urol (2014) 66:1157–64. 10.1016/j.eururo.2014.02.042 24630414

[34] Yuk HD Jeong CW Kwak C Kim HH Ku JH. Elevated Neutrophil to Lymphocyte Ratio Predicts Poor Prognosis in Non-Muscle Invasive Bladder Cancer Patients: Initial Intravesical Bacillus Calmette-Guerin Treatment After Transurethral Resection of Bladder Tumor Setting. Front Oncol (2018) 8:642. 10.3389/fonc.2018.00642 30705874 PMC6344445

