# Supplementary material for: A Novel Nomogram Based on Machine Learning-Pathomics Signature and Neutrophil to Lymphocyte Ratio for Survival Prediction of Bladder Cancer Patients
Source: Front Oncol. 2021 Jun 17;11:703033. doi: 10.3389/fonc.2021.703033 (PMC8247435; doi:10.3389/fonc.2021.703033)
Supplement: Supplementary file 1 [file Table_1.docx]

**Table S1.** Comprehensive list of quantitative features extracted by the BCa image processing pipeline.

| **NO.** | **Item** |
| --- | --- |
| 1 | Cell: Area |
| 2 | Cell: Circularity |
| 3 | Cell: Eccentricity |
| 4 | Cell: Eosin OD max |
| 5 | Cell: Eosin OD mean |
| 6 | Cell: Eosin OD min |
| 7 | Cell: Eosin OD std dev |
| 8 | Cell: Hematoxylin OD max |
| 9 | Cell: Hematoxylin OD mean |
| 10 | Cell: Hematoxylin OD min |
| 11 | Cell: Hematoxylin OD std dev |
| 12 | Cell: Max caliper |
| 13 | Cell: Min caliper |
| 14 | Cell: Perimeter |
| 15 | Centroid X |
| 16 | Centroid Y |
| 17 | Cytoplasm: Eosin OD max |
| 18 | Cytoplasm: Eosin OD mean |
| 19 | Cytoplasm: Eosin OD min |
| 20 | Cytoplasm: Eosin OD std dev |
| 21 | Cytoplasm: Hematoxylin OD max |
| 22 | Cytoplasm: Hematoxylin OD mean |
| 23 | Cytoplasm: Hematoxylin OD min |
| 24 | Cytoplasm: Hematoxylin OD std dev |
| 25 | Nucleus/Cell area ratio |
| 26 | Nucleus: Area |
| 27 | Nucleus: Circularity |
| 28 | Nucleus: Eccentricity |
| 29 | Nucleus: Eosin OD max |
| 30 | Nucleus: Eosin OD mean |
| 31 | Nucleus: Eosin OD min |
| 32 | Nucleus: Eosin OD range |
| 33 | Nucleus: Eosin OD std dev |
| 34 | Nucleus: Eosin OD sum |
| 35 | Nucleus: Hematoxylin OD max |
| 36 | Nucleus: Hematoxylin OD mean |
| 37 | Nucleus: Hematoxylin OD min |
| 38 | Nucleus: Hematoxylin OD range |
| 39 | Nucleus: Hematoxylin OD std dev |
| 40 | Nucleus: Hematoxylin OD sum |
| 41 | Nucleus: Max caliper |
| 42 | Nucleus: Min caliper |
| 43 | Nucleus: Perimeter |
